# Linagliptin, when compared to placebo, improves CD34+ve endothelial progenitor cells in type 2 diabetes subjects with chronic kidney disease taking metformin and/or insulin: a randomized controlled trial

**DOI:** 10.1186/s12933-020-01046-z

**Published:** 2020-06-03

**Authors:** Hassan B. Awal, Seshagiri Rao Nandula, Cleyton C. Domingues, Fiona J. Dore, Nabanita Kundu, Beda Brichacek, Mona Fakhri, Adrian Elzarki, Neeki Ahmadi, Shauna Safai, Magan Fosso, Richard L. Amdur, Sabyasachi Sen

**Affiliations:** 1 The GW Medical Faculty Associates, 2300 M Street NW, Washington, DC 20037 USA; 2 Department of Medicine, The George Washington University, 2300 I St NW, SMHS Room 462, Washington, DC 20052 USA

**Keywords:** Linagliptin, EPC, Endothelium, CD34+, Type 2 diabetes, Diabetic kidney disease

## Abstract

**Background:**

Endothelial Progenitor cells (EPCs) has been shown to be dysfunctional in both type 2 diabetes mellitus (T2DM) and chronic kidney disease (CKD) leading to poor regeneration of endothelium and renal perfusion. EPCs have been shown to be a robust cardiovascular disease (CVD) risk indicator. Cellular mechanisms of DPP4 inhibitors such as linagliptin (LG) on CVD risk, in patients with T2DM with established CKD has not been established. Linagliptin, a DPP4 inhibitor when added to insulin, metformin or both may improve endothelial dysfunction in a diabetic kidney disease (DKD) population.

**Methods:**

31 subjects taking metformin and/or Insulin were enrolled in this 12 weeks, double blind, randomized placebo matched trial, with 5 mg LG compared to placebo. Type 2 diabetes subjects (30–70 years old), HbA1c of 6.5–10%, CKD Stage 1–3 were included. CD34+ cell number, migratory function, gene expression along with vascular parameters such as arterial stiffness, biochemistry, resting energy expenditure and body composition were measured. Data were collected at week 0, 6 and 12. A mixed model regression analysis was done with p value < 0.05 considered significant.

**Results:**

A double positive CD34/CD184 cell count had a statistically significant increase (p < 0.02) as determined by flow cytometry in LG group where CD184 is SDF1a cell surface receptor. Though mRNA differences in CD34+ve was more pronounced CD34- cell mRNA analysis showed increase in antioxidants (superoxide dismutase 2 or SOD2, Catalase and Glutathione Peroxidase or GPX) and prominent endothelial markers (PECAM1, VEGF-A, vWF and NOS3). Arterial stiffness measures such as augmentation Index (AI) (p < 0.04) and pulse wave analysis (PWV) were improved (reduced in stiffness) in LG group. A reduction in LDL: HDL ratio was noted in treatment group (p < 0.04). Urinary exosome protein examining podocyte health (podocalyxin, Wilms tumor and nephrin) showed reduction or improvement.

**Conclusions:**

In DKD subjects, Linagliptin promotes an increase in CXCR4 expression on CD34 + progenitor cells with a concomitant improvement in vascular and renal parameters at 12 weeks.

*Trial Registration Number* NCT02467478 Date of Registration: 06/08/2015

## Background

Over 1 in 10 Americans are suffering from Type 2 Diabetes, which in recent times has risen to a national epidemic [1, 2]. Diabetes and chronic kidney disease (CKD) are conditions that are responsible for vascular damage and complications, both micro and macrovascular, including endothelial dysfunction, endothelial cell inflammation, oxidative stress, and cardiovascular pro-thrombotic states [3–5]. Endothelial progenitor cells (EPCS, defined here as CD34 + cells) are specialized stem cells responsible for repair of the endothelial cell lining of blood vessels and angiogenesis. These cells can be harvested from peripheral whole blood derived mononuclear cells (MNC) by positive sorting for CD34 peripheral cell surface receptor. It has been shown that high glucose environment, as seen in diabetes mellitus (either type 1 or 2), leads to functional impairment of circulating EPCs. Their number goes down, along with their ability to form colonies and migration to the site of endothelial damage [6–10]. Other studies have shown that healthy CD34 + cells can effectively repair damaged endothelial cell lining [11].

We currently use serum based biochemical parameters for estimation of cardiovascular disease (CVD) risk, which might take several weeks or even months [12] to change as they are paracrine properties of a particular cell lines that is injured or damaged, such as hs-CRP, a factor produced from inflamed endothelium. Based on literature and our past CD34+ve cell based studies, it is likely that circulating CD34+ EPC number, function and mRNA expression can act as a robust cellular biomarker that is more reliable than serum-based biomarkers for monitoring endothelial dysfunction in type 2 diabetes [13–17].

Dipeptidyl peptidase-4 (DPP-4) enzyme inhibitors, are a class of oral anti-diabetic medications, that have been shown to achieve improved glycemic control by lowering HbA1C, without causing hypoglycemia, and are weight neutral [18]. DPP-4 enzyme degrades incretins such as GLP1 and GIP, including chemotactic factors such as SDF-1ɑ (stromal derived factor). Therefore, use of DPP4 inhibitor is expected to be associated with increased bioavailability of SDF1a. This may help “homing-in” of CD34+ endothelial progenitor cells to the damaged endothelial sites (that are producing SDF1a) thereby helping endothelial regeneration. This can be a potential mechanism to prevent endothelial damage which may translate to vascular damage repair through-out the body. However, there is limited data demonstrating the potential cardiovascular effect of these medications. A few studies using either Sitagliptin or Saxagliptin have shown an increase in endothelial progenitor cells, and thus potential cardiovascular benefits, with DPP-4 therapy is possible [13, 14, 19]. Literature has shown that DPP-4 inhibitors may increase EPC mobilization from the bone marrow by increasing SDF-1α in the plasma [13, 20]. The upregulation of SDF1α and also VEGF in the plasma increase mobilization and recruitment of EPCs to the site of the ischemic injury for repair and regeneration [21–24].

As mentioned before chronic kidney disease (CKD) is a CVD equivalent risk factor independent of diabetes. It is unknown whether Linagliptin, a DPP-4 inhibitor, will have any positive effect on human EPC function with two prominent cardiovascular risk factors co-existing such as CKD and T2DM.

In this 12-week placebo-matched clinical trial, we studied the effect of Linagliptin. Linagliptin was added to metformin and/or Insulin, in subjects with type 2 diabetes and Stage I–III chronic kidney disease but without any established adverse cardiovascular event (such as history of myocardial infarction or cerebral stroke).

## Methods

### Trial design and oversight

This is a phase 4 (post-marketing), two arm, single site, parallel group, double blind, placebo controlled randomized clinical trial comparing Linagliptin 5 mg tablets, taken orally, once daily, with matching placebo. The study was conducted in accordance with good clinical practice guidelines set forth by the International Conference of harmonization and any local regulatory guidelines with the approval and oversight of the George Washington University Institutional Review Board. The trial was funded by Boehringer Ingelheim as an Investigator Initiated Study (IIS) and conducted by the Investigator-Sponsor Sabyasachi Sen, MD, at the George Washington University.

Subjects were initially pre-screened to assess eligibility. Once determined preliminary eligibility, they were brought in for a screening visit to confirm eligibility via interview, medical record check and laboratory workup once the subject signed the informed consent. The subjects were then enrolled into one of two arms of the study: 5 mg Linagliptin or matching placebo.

17 subjects were enrolled into the active group and 14 subjects were enrolled into the placebo group.

Subjects were randomized using a permuted block design, developed by the epidemiology and biostatistics research core. This approach ensures groups will be approximately balanced at any time during the study and completion. This approach is similar to our published method in a similar study using saxagliptin [25].

There were 3 study visits in total, first at week 0, second at week 6 and third at week 12. All three visits had the same assessments. The assessments that were done were: vital measurements, adverse event (AE) check and a peripheral blood draw. Approximately 80 ml of blood was drawn for CD34+ endothelial progenitor cell harvesting and routine blood work.

Other parameters tested were resting metabolic rate (RMR, energy expenditure), measurement of waist to hip ratio, urine sample collection, Tanita body composition scale, pulse wave analysis and pulse wave velocity to determine arterial stiffness. Subjects were advised to adhere to 150 min of weekly aerobic exercise and their activity levels were monitored using ACTi graph activity monitor.

A follow up phone call visit was done 30 days from the last in-person visit to assess for any residual adverse events (AE).

### Participants

Subjects were included if they were between 30 and 70 years old inclusive, with a diagnosis of T2DM for 15 years or less. Glycated hemoglobin level (HbA1c) inclusions were between 6.5 and 10.0% Inclusive. Their baseline medications were stable dose of Insulin (either short acting or long acting) and/or Metformin (1–2 g/day). A stable dose was considered to be at least the maximum labeled dose or dose not associated with unacceptable side effects. Patients with BMI between 25 and 39.9 kg/m^2^ were included, thereby excluding severe obesity. Only, patients with impaired renal function were included, with Chronic Kidney Disease stage 1 to 3, defined as estimated minimum GFR of 30 ml/min/1.73 (GFR, as calculated by MDRD formula).

Any patients with Type I diabetes, history of Diabetic ketoacidosis, low hematocrit (less than 28 units), history of recent pancreatitis or cancer, recent coronary or cerebrovascular event within 6 months, use of consistent steroid medications, untreated thyroid disease was excluded.

Additional inclusion and exclusion criteria can be found in Appendix 1. Baseline Characteristics are mentioned in Table 1.

**Table 1:** Baseline characteristics and demographics

Variable	Placebo (n = 17)	Linagliptin (n = 14)	P
Age (years), mean ± SD	63 ± 6	61 ± 5	0.21
Sex female, n %	10 (59%)	3 (21%)	0.04
Race			0.49
BL	12 (71%)	8 (57%)	
Wh	4 (24%)	3 (21%)	
Other	1 (6%)	3 (21%)	
Medications			
Metformin	14 (82%)	12 (86%)	0.99
Insulin	6 (35%)	5 (36%)	0.99
BP			
Systolic	133 ± 18	128 ± 10	0.30
Diastolic	77 ± 7	81 ± 7	0.23
BMI	30.6 ± 2.9	31.2 ± 4.4	0.67
Percent fat	38 ± 10	30 ± 10	0.04
Waist cm	105 ± 8	107 ± 17	0.22
Basic metabolic rate	1476 ± 448	1868 ± 293	0.02
Fasting glucose	130 ± 44	125 ± 26	0.70
Serum creatinine	0.9 ± 0.3	1.1 ± 0.4	0.24
eGFR	84 ± 19	83 ± 21	0.85
Cholesterol	168 ± 53	166 ± 52	0.92
HbA1c	7.4 ± 1.0	7.1 ± 0.7	0.50

### Outcome objectives

The primary objective is to ascertain if addition of Linagliptin improves CD34+ cell number (CD34+ number, %CD34+ of total Mononuclear Cell population) function (cell migration function in response to SDF1α) and gene expression, in T2DM with CKD Stages 1–3, which will be correlated to improvement in 24 h of urinary protein estimation and serum creatinine clearance.

The secondary objective is to correlate the cellular outcome measures with other measures of endothelial function such as Arterial Stiffness [measured by pulse wave analysis (Augmentation Index) and pulse wave velocity (m/s)], serum biochemistry (complete metabolic panel or CMP, interleukin-6 or IL6, highly sensitive C-reactive protein or hsCRP, Leptin, serum insulin, TNFα), adiposity (as % body fat), resting energy expenditure, REE (in kcal) and glycemic control (through HbA1c).

### Cellular and clinical assessments

#### CD34+ endothelial progenitor cell analysis

Peripheral blood samples (approximately 60 ml) were drawn from patients and phosphate buffered saline (1:1) was added. Identification and quantification of circulating cell phenotypes was performed on fresh blood samples, within 3 h after collection, using flow cytometry. Briefly, mononuclear cells (MNCs) were then isolated from whole blood using a Ficoll density centrifuge method. MNCs were counted and aliquot was used for CFU-Hill colony formation assay following the manufacturer’s instruction (Stem Cell Technologies, Vancouver, BC, Canada). Colony forming unit (CFU) was counted at day14. A fraction of the MNC were stained with fluorescein isothiocyanate (FITC)-conjugated antihuman CD34, Allophycocyanin (APC) conjugatedantihuman CD184 (CXCR4) and FITC conjugated antihumanCD31 antibodies (Miltenyi Biotec GmbH, Bergisch-Gladback, Germany) in order to analyze specific progenitor cell surface marker (CD34) and mature endothelial cell surface markers (CD31) or receptor for SDF1a ligand, CXCR4) by flow cytometry. After gating mononuclear cells in the side scatter (SSC)-A vs forward scatter (FSC)-A plot, CD34/CD184 single- and double-positive cells were identified. Cells were acquired on a fluorescence-activated cell sorter (FACS) Canto instrument (Becton–Dickinson) and scored with the FloJo software.

To isolate EPCs (CD34+), MNCs were magnetically sorted through a column after cells were stained with CD34+ microbeads antibody (Miltenyi Biotec GmbH, Bergisch Gladback, Germany). An aliquot of CD34+ cells were then stained with trypan blue and counted using an Auto Cellometer Mini (Nexcelom Bioscience, Lawrence, MA) to assess viability.

CD34+ gene expression analysis was performed by quantitative reverse transcriptase polymerase chain reaction (qRT-PCR) as previously described [25]. CD34+ve cell total mRNA was extracted and purified using the RNeasy Minikit (Qiagen, Germany). mRNA was then converted into cDNA by using the high capacity cDNA reverse transcriptase kit (Thermo Fisher Scientific, MA) Possible gene expression changes promoted by Linagliptin was assessed by a CFX96 real-time PCR systems (Bio-Rad, CA.) using Taqman Universal masters Mix II (Thermo Fisher Scientific, MA) and inventoried probes. The gene expression analysis included antioxidants, apoptosis, endothelial functions, chemotaxis, inflammation and endothelial lineage cell surface markers. The expression of each individual gene was normalized to either housekeeping 18S or GAPDH and calculated using C-ddct method considering the difference in cycle threshold between visit 2 and 3 and baseline (Visit 1). mRNA gene expression of CD34-cell population (from MNC population) was also analyzed along with CD34+ cells.

Our methods are similar to our published method in a similar study using saxagliptin [25].

The migratory capacity of CD34+ was evaluated using the CytoSelect 24-well Cell Migration Assay kit (Cell Biolads, Inc., San Diego, CA). Cells were suspended in Serum free media and seeded at 100,000 cells per insert. Migration of the cells through a 3 um polycarbonate membrane to the wells containing a serum-free media (control) and chemoattractant SDF-1α (10 or 100 ng/mL) was assessed after cells were kept overnight in incubator. Migratory cells were dissociated from the membrane and subsequently lysed and quantified by fluorescence (480 nm/530 nm) using CyQuant GR dye (Cells Biolabs, Inc, San Diego, CA). The fluorescence ratios between cells exposed to the chemotactic factor and cells exposed to chemoattractant-free media (control) along the visits were used to analyze the migratory capacity of the cells.

As is the case with diabetic patients, number of isolated CD34+ cells are usually not as high as anticipated due to established endothelial damage and because the progenitor cells are very susceptible to apoptotic death in hyperglycemia. Hence, in order to understand some of the effect of protein upregulation we have done the analysis on all remaining CD34− cells as they are the remaining 99% of Polymorphonuclear cells. The rationale was that any gene expression upregulation on CD34+ cells should also be prevalent in CD34− cells and should by and large reflect the mRNA analysis of unsorted MNC population.

#### Body composition measurement

Body composition measurement was carried out using Tanita™ BF-350 Body Composition Scale and manually. Manual measurement included height, waist circumference, hip circumference. Tanita scale uses a bio-impedance electrical impulse to measure body fat percent, fat mass (kg), fat free mass (kg), percent body water, water mass (kg) alongside weight. It then calculates the BMI and estimated basal metabolic rate.

#### Basal metabolic rate measurement

Resting energy expenditure (REE) was measured using KORR REEVUE. Test was conducted with the subject sitting and well rested. Subject was instructed to keep a tight seal around the mouthpiece and use the nose clip to avoid breathing in from the nose. The test ran for about 10 min. It calculated estimated REE, predicted REE, estimated TEE (Total Energy Expenditure), VO2 Max and estimated calorie intake per day.

#### Arterial stiffness

This parameter was measured using AtCor SphygmoCor CP system. We obtained two outcomes such as: Pulse Wave Velocity and Pulse Wave Analysis. The patient was supine on the examination table, 3 leads were attached on right forearm, left forearm and left shin.

Pulse wave analysis (PWA) was measured on the left Radial Artery with the subject supine. At least three readings were taken with operator index ≥ 80. Measurement includes augmentation index (AI), Augmentation Index adjusted for Heart Rate of 75 (AI-75), Augmentation Pressure (AP), Aortic and Radial reading of systolic, diastolic, pulse pressure and mean pressure.

Pulse wave velocity (PWV) was measured with the subject in supine position. This measurement requires a distal and proximal artery. Distal was used as right femoral artery with proximal being the left carotid. Index and ring fingers were used to manually localize the pulse, sometimes an arterial Doppler was used to localize the femoral pulse on patient with challenging body habitus. Once a stable pulse waveform was observed, the probe position was kept stable for 20 more pulses before the reading was finalized. Three readings were taken with standard deviation of less than 10%. The result reported a velocity in m/s, alongside the standard deviation with error.

#### Biological sample and vital collection

A venous blood sample was collected from the Antecubital fossa. About 80 ml of blood was collected. 60 ml for EPC analysis and 20 ml for standard of care blood works which included Basic Metabolic Panel, Lipid Panel, HbA1c, hsCRP, IL6, Adiponectin and Insulin. ELISA was performed to analyze serum GLP1 and SDF1α using ELISA Immunoassay kit (Raybiotech, Norcross, GA) for GLP1 and Sandwich ELISA (EHCXCL12A, Thermo Scientific) for SDF1α. Urine sample was collected for urine Microalbumin and Creatinine ratio. Vitals were gathered on the left arm, systolic pressure, diastolic pressure and heart rate, along with sublingual temperature.

#### ACTi graph activity monitor

Subjects level of activity was measured using Actigraph wGT3x-BT activity monitors. Subjects was advised on diet and exercise instructed to wear the meter during all waking hours and was advised to adhere to 150 min of moderate intensity aerobic exercise per week. Actigraph served as a measure of this exercise compliance, and to verify for exercise as a confounding variable.

#### Urine exosome analysis: Polyethylene glycol (PEG) enrichment of extracellular vesicles

The cells debris and large apoptotic bodies were removed from the urine samples by centrifugation at 500*g* for 5 min followed by 3000*g* for 30 min at 4° C. Transfer supernatant into ultracentrifugation tubes and centrifuged at 100,000*g* at 4° C for 75 min (Optimal XPN-100 centrifuge, Beckmann Coulter Inc, US). After ultra-centrifugation the pellet was dissolved in RIPA buffer with protease inhibitor cocktail and stored the sample at -80° C for further analysis.

Western blotting: Extracellular vesicle extracts were fractionated by SDS-PAGE and transferred to a polyvinylidene difluoride membrane using a transfer apparatus according to the manufacturer’s protocols (Bio-Rad). After incubation with 5% nonfat milk in TBST (10 mM Tris, pH 8.0, 150 mM NaCl, 0.5% Tween 20) for 60 min. The membrane was washed once with TBST and incubated with antibodies against CD9 (1:1000), CD81 (1:1000), CD63 (1:1000), HSP70 (1:1000), anti-podocalyxin (PODXL, 1:1000), anti-Wilms tumor protein (1:1000) and anti-nephrin antibody (1:1000) at 4 °C for 12 h. Membranes were washed three times for 10 min and incubated with a 1:20,000 dilution of horseradish peroxidase-conjugated goat anti-rabbit antibody for 90 min at room temperature. Blots were washed with TBST three times and developed with Pierce ECL kit (ThemoFisher Scientific, USA).

### Statistical analysis

Power calculation: This is a pilot study and accurate power calculation is not feasible.

The effect of a single session, as well as extended training, on healthy subjects or those with existing cardiovascular conditions appears to increase the CD34+/KDR+ cells and VEGF. To compute sample size we used the approach suggested by Diggle, Liang, and Zeger which compares the rates of change in the two study groups over time. This approach incorporates the number and interval of time points and the correlation among repeated measures. For this study, we will employ one baseline and two follow-up measures at 6 and 12 weeks. Further, we will assume a correlation 0.60 among repeated measures of the outcome. We consider this a conservative estimate since Frison and Pocock suggest a correlation of 0.65 as reasonable in the absence of an existing estimate. We also note that as this correlation increases, statistical power also increases.

The results in the table below show the expected mean difference in study groups at the end of follow-up, as well as the average rate of change in the two groups at 80% power and 90% power. To estimate the effect of Linagliptin on the CD34+/KDR+ cells, we expect that the effect would be at least 25% greater than the effect seen for exercise alone. Using the results from Sandri et al. for the rate of change and the variability, the CD34+/KDR+ cells increased an average rate of about 4/week with a standard deviation of about 15. Thus, for a 25% increase in the rate of change for the CD34+/KDR+ cells due to Linagliptin, a sample size of 18 subjects per group would provide about 84% power, assuming measures taken at baseline and 2 equally-spaced time points over 12 weeks. At the conclusion of follow-up, we would expect study groups to differ by an average of 12 cells. If the effect of Linagliptin is only 20%, a sample of 18 would provide about 70% power, whereas a sample of 20 would provide about 73% power.

Thus, we feel that a sample size of at least 18 subjects per group with complete data would provide sufficient power for the study outcomes. In order to ensure that we will have 18 per group who complete the study, we will enroll 20 subjects per group in order to account for attrition over the 12-week intervention period.

**Table:** 

Biochemical measure	Mean difference at end of 12 weeks	Sample size per group	Power
CD34+/KDR+ cells	12 cells (25% increase, 4/week)	18	0.84
	vs 5/week	22	0.90

Analysis: Continuous variable distributions were examined using histograms for skewness or outliers. When these were present, we did not use parametric statistics for these variables. For normal variables, we used 2-tailed between-groups t-tests to examine differences between treatment groups at baseline on continuous variables, and either Chi square or the Fishers Exact test for categorical variables. To examine differences between treatment groups across all time points, as well as time effects, and whether the slope of change over time differed between treatments, we used random effects mixed model regression, examining the main effects of treatment (Linagliptin vs placebo), and time (v1, v2, v3), and the treatment by time interaction. This method allows us to use all non-missing subject data and adjusts for within-subject auto-correlation. For variables with significant effects in the mixed models, we examined the means graphically. For skewed variables or those with outliers, we used the Kruskal–Wallis test to examine differences in the distribution location (i.e. median) within time points, across treatment groups. SAS (version 9.4, Cary, NC) was used for data analysis with p < 0.05 considered significant.

Since subjects were randomized to treatment, chance of baseline subject characterizations acting as confounders are minimized. Therefore, randomized control trials do not usually adjust for baseline differences. In gene expression variables, outliers with expression values > 50 were dropped, and values were natural log transformed due to skewness [using log (expression +1)]. Expression values that still had outliers after log transformation were capped at value of 2.

## Results

### Primary outcome

**Fig. 1:**
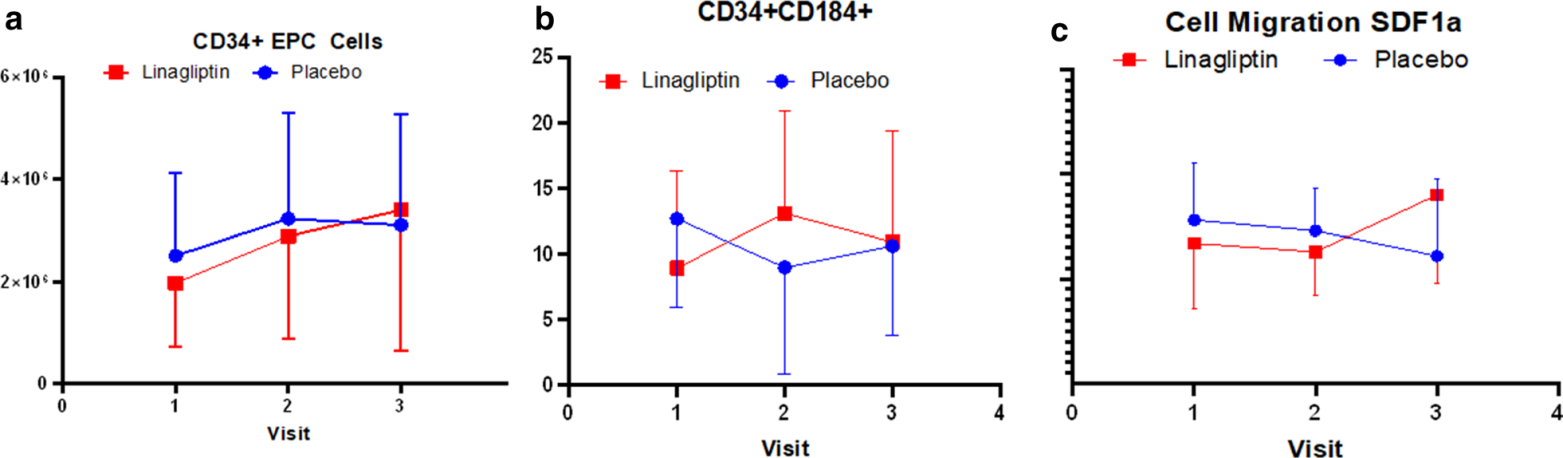
Endothelial progenitor (CD34+), CD34+ CD184+ cell expression and Migration. Flow cytometry-based assay for CD34positive cells and CD34+ CD184 dual positive cells. **a** Mean CD34 cell number increased in Linagliptin group from visit 1 to 3. **b** The functional improvement of CD34+ cells can be further supported by statistically significant expression of CD34+ CD184 dual positive cells (CXCR4) (*p *< 0.02) measured by flow cytometry. Interesting pattern can be appreciated here, as double positivity dropped from visit 1 to 2 in the placebo group but in Linagliptin group it went up, though the graphs merged at visit 3. **c** The mean fluorescence intensities (normalized to control samples) is shown here. A trend in increased migratory response of CD34+ve cells to the chemotactic factor SDF1α(100 ng/ml) is observed in linagliptin group at visit 3

Characterization of endothelial progenitor cells (CD34+ve): To find out the effect of DPP4 inhibitor, linagliptin, on the endothelial progenitor cell number, we counted the total CD34+ cell number in both placebo and linagliptin groups. The flowcytometric analysis of the cell numbers as shown in the Fig. 1a, the number of cells purified from MNCs did not show any significant difference between the groups, at visit 3, however, the CD34+ cell number increased from visit 1 to 3 in linagliptin group. Mean CD34+ CD184+ double positive cells increased from visit 1 to 2 in linagliptin group as compared to the placebo group (Fig. 1b).

The migratory response of CD34+ cells to the chemotactic factor SDF1α (100 ng/ml l) was not statistically significant between the groups. However, the migratory response to 100 ng/ml of SDF1α showed a trend in increase in the linagliptin group starting at visit 2 (Fig. 1c). That is why there was a steep rise in migration of CD34+ve cells in response to SDF1 α between visit 2 and 3, in the linagliptin group.

#### Gene expression analysis

##### The effect of linagliptin on the gene expression of CD34 negative cells

**Fig. 2:**
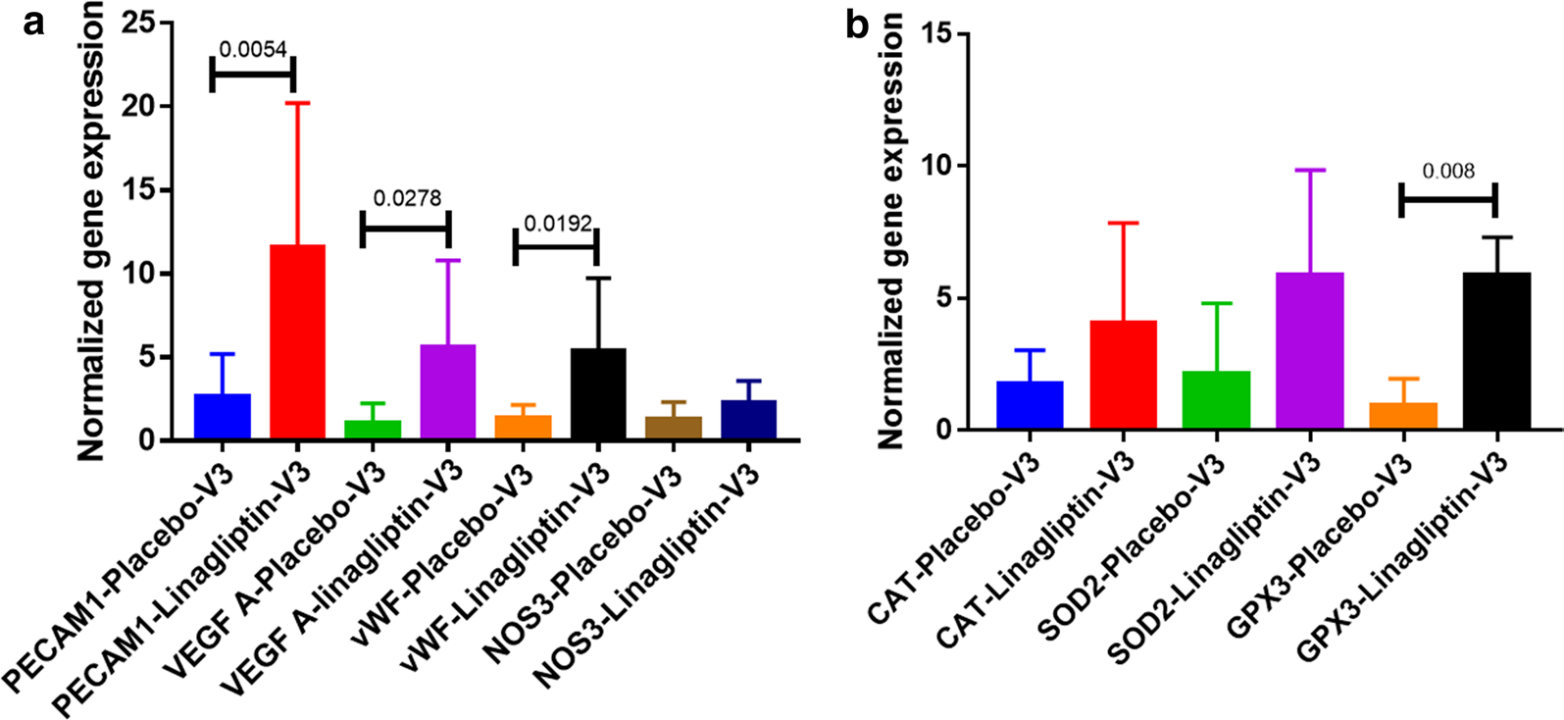
Gene expression of endothelial markers and antioxidant increased significantly. **a** Endothelial markers PECAM1, VEGF-A and vWF gene expression on CD34 negative cells from both placebo and Linagliptin Visit 3 patients. vWF mRNA expression is predominantly increased in at Visit-3 in Linagliptin group. Gene expression is normalized with to 18S and values are related to Visit-1. **b** Gene expression of antioxidant markers such as Catalase(CAT), Superoxide dismutase-2 or MnSOD (SOD2) and glutathione peroxidase (GPX3) gene expression on CD34 negative cells from both placebo and Linagliptin Visit 3 patients, shown here. mRNA expressions of these genes are increased in at Visit-3 in the Linagliptin group. Gene expression is normalized to 18S and gene expression values are fold difference to visit-1 values

**Fig. 3:**
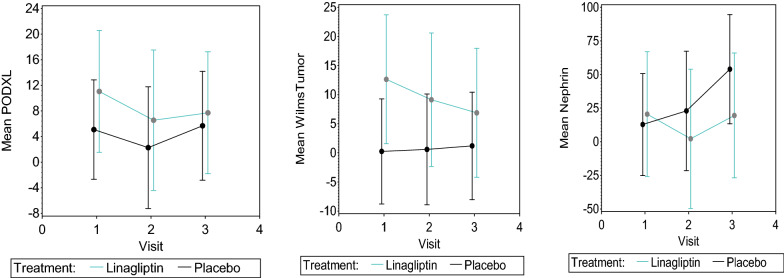
Urinary exosomal markers podocalxyin, Wilm’s tumor and Nephrin identified by Western blot. Urinary exosomal proteins noted in urine where increase in protein amount indicates worsening podocyte health were improved (or reduced) in linagliptin group in all three markers, from visit 1 to 3 whereas the trend was a flat line or increased in the placebo. Mean band intensities were represented for both placebo and Linagliptin groups from visit 1, 2, and 3. Exosomal protein CD9 was used for normalization. Line graphs for individual proteins noted in urinary exosomes are shown

Gene expression analysis was performed for antioxidants (SOD2, GPX1, CAT), endothelial function (VEGFA, PECAM1, eNOS) and endothelial cell lineage surface marker. Due to insufficient mRNA isolation from low number of CD34+ cells, gene expression analysis was not helpful. We also looked at CD34-ve cells which is expected to be similar to unsorted MNC population. The gene expression analysis by qPCR for endothelial markers PECAM1, VEGF-A and vWF has increased 11 fold, fivefold and fivefold simultaneously in the linagliptin group in visit3 (Fig. 2a). A twofold upregulation of these genes’ mRNA expression in visit 2 is also been observed (data not shown). Since the endothelial gene expression is increased significantly, we were interested to see the expression of endothelial functional genes vWF and NOS3(or endothelial nitric oxide synthase, eNOS). Both were upregulated by fivefold and twofold in linagliptin group in visit 3 (Fig. 3).

Later we also analyzed the mRNA expression of antioxidant genes that are known to play a key role in cellular redox balance, such as superoxide dismutase 2 (SOD2), catalase (CAT) and glutathione peroxide 1 (GPX-1). Again, we have observed a marked increase in expression of these genes in the CD34 negative cells from linagliptin group in visit 3 as shown in Fig. 2b.

### Urinary function marker

#### Quantification of exosomal proteins in urine samples by western blot

Recently urinary exosomes are being used as biomarker for kidney diseases. We were interested to study urinary exosomal expression for nephrin, renal Wilm’s Tumor (WT-1) and podocalyxin like protein 1(PODXL) in urine samples from placebo and linagliptin group. As shown in the Fig. 3, the band intensities for the PODXL and WT-1 were high in visit 1 in linagliptin group as compared to placebo group. Whereas, there is no difference in band intensities for the PODXL and WT-1 proteins in Visit 2 and 3 between the groups. Nephrin expression was not different between the groups in visit 1 and 2 where as in visit 3 the placebo group has shown more Nephrin band intensity as compared to linagliptin group.

**Fig. 4:**
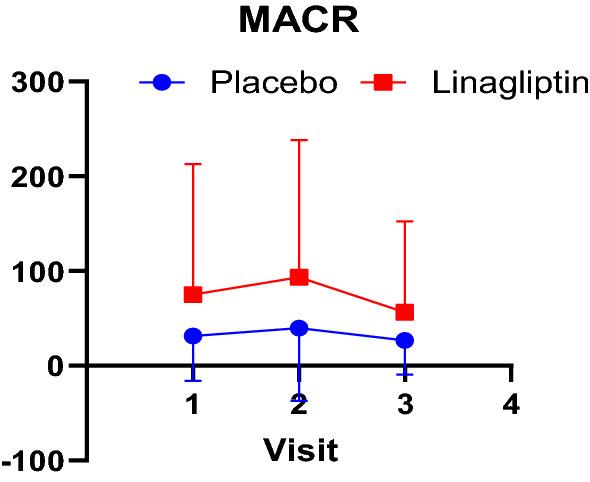
Microalbumin over Creatinine ratio. Microalbumin: Creatinine ratio shown as line graphs over three visits. Between visit 1 and 2, the Lina and placebo line trajectories are similar, however between visit 2 and 3, there is a sharp drop in the line trajectory particularly for Lina group. This may indicate improvement in renal function with Linagliptin, gradually over a 12-week period. This observation (p value) was not statistically significant

There was no significant change in the eGFR and serum creatinine between the two groups however interesting pattern can be appreciated when one plots the change in urine microalbumin over creatinine ratio (Fig. 4). At baseline the linagliptin group had higher urine proteinuria and that remained stable from visit 1 to visit 2. From visit 2, proteinuria started to drop in both groups, but the reduction gradient was more acute and obvious in the linagliptin group.

### Secondary objective measures

#### Venous blood biochemistries

Venous blood biochemistries were gathered, both through Labcorp of America and through serum ELISA and both standard of care and research labs were collected.

**Fig. 5:**
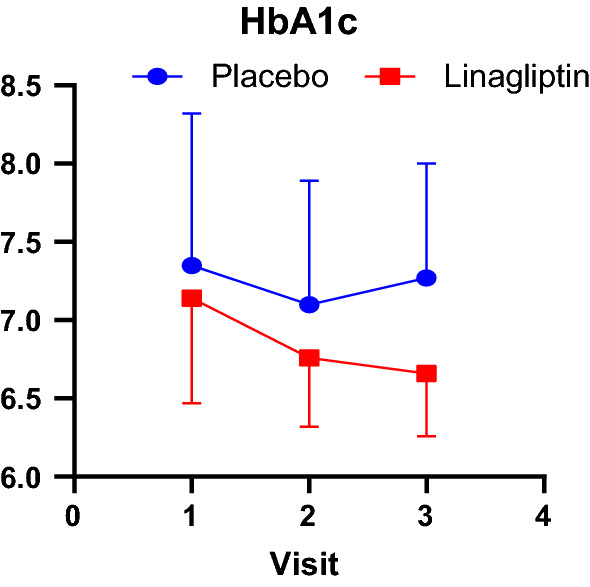
HbA1c: The strongest effect of all the variable is the effect on time on HbA1c (p < 0.005), which means HbA1c went down substantially in both the treatment and control group. Even though the effect of treatment on this variable was not significant after the effect of time was accounted for, interesting trend pattern can be appreciated when it’s graphed. The Hba1c is relatively stable in the placebo group but has a clear downward trend in the treatment group

Detailed lab values of selected significant parameter are on Table 2. We found statistically significant difference in the LDL over HDL ratio (p < 0.04). The strongest effect of all the variable is the effect on time on HbA1c (p < 0.005), which means HbA1c went down substantially in both the treatment and control group (Fig. 5). Even though the effect of treatment on this variable was not significant after the effect of time was accounted for, interesting trend pattern can be appreciated when it’s graphed. The Hba1c is relatively stable in the placebo group but has a clear downward trend in the treatment group.

**Table 2:** Blood biochemistry before and after linagliptin treatment

	Visit 1	Visit 2	Visit 3	*P* value
Glucose				0.47
Placebo	129.88 ± 43.78	129.35 ± 41.39	129.65 ± 46.78	
Linagliptin	124.71 ± 25.94	116.15 ± 23.16	109.92 ± 16.81	
BUN				0.07
Placebo	16.00 ± 5.56	17.41 ± 5.98	16.76 ± 5.85	
Linagliptin	18.07 ± 7.33	16.08 ± 6.10	16.85 ± 6.41	
Serum creatinine				0.59
Placebo	0.93 ± 0.27	0.91 ± 0.24	0.92 ± 0.25	
Linagliptin	1.06 ± 0.35	1.08 ± 0.43	1.1 ± 0.38	
eGFR				0.78
Placebo	84.06 ± 19.41	85.88 ± 19.41	84.12 ± 19.81	
Linagliptin	82.71 ± 20.96	82.85 ± 22.38	79.46 ± 19.9	
Cholesterol				0.14
Placebo	168.12 ± 53.16	173.29 ± 40.41	171.65 ± 48.49	
Linagliptin	166.29 ± 51.63	154.46 ± 37.32	159.69 ± 48.53	
Triglycerides				0.13
Placebo	118 ± 81.86	143.47 ± 126.7	127.76 ± 79.04	
Linagliptin	133.43 ± 67.97	130 ± 58.64	124.31 ± 50.67	
LDL/HDL				0.04
Placebo	1.76 ± 1.00	1.82 ± 0.83	1.76 ± 0.89	
Linagliptin	1.83 ± 0.78	1.65 ± 0.7	1.92 ± 0.87	
HbA1c				0.12
Placebo	7.35 ± 0.97	7.10 ± 0.79	7.27 ± 0.73	
Linagliptin	7.14 ± 0.67	6.76 ± 0.44	6.66 ± 0.40	
C-reactive protein				0.49
Placebo	4.50 ± 9.73	2.77 ± 2.51	3.08 ± 3.41	
Linagliptin	13.53 ± 31.32	8.4 ± 16.03	5.17 ± 6.54	
IL6				0.18
Placebo	5.16 ± 10.71	2.72 ± 3.01	2.18 ± 1.45	
Linagliptin	6.42 ± 11.00	5.24 ± 7.39	5.09 ± 6.08	
TNFα				0.71
Placebo	4.01 ± 10.42	5.94 ± 12.13	1.77 ± 1.94	
Linagliptin	4.10 ± 7.4	2.25 ± 2.57	1.92 ± 1.52	
Leptin				0.43
Placebo	22.48 ± 12.6	54.08 ± 130.71	22.88 ± 13.25	
Linagliptin	21.69 ± 22.77	23.06 ± 25.72	23.91 ± 25.83	
Adiponectin				0.98
Placebo	6.57 ± 5.04	6.5 ± 6.59	6.64 ± 6.33	
Linagliptin	5.39 ± 3.08	5.52 ± 2.98	5.45 ± 3.28	
Insulin				0.87
Placebo	17.85 ± 14.07	19.84 ± 11.83	20.82 ± 12.6	
Linagliptin	16.43 ± 12.9	16.06 ± 8.51	20.52 ± 19.71	
Uric acid				0.97
Placebo	6.51 ± 1.57	6.59 ± 1.21	6.73 ± 1.43	
Linagliptin	5.89 ± 1.78	5.96 ± 1.49	12.52 ± 21.85

#### Arterial stiffness

Stiffness of an artery significantly contributes to lack of pliability and contractility and is an important marker of increased peripheral resistance, diastolic dysfunction and systemic hypertension. It is associated with cardiovascular diseases in older individuals and is positively associated with hypertension, coronary artery disease, stroke, heart failure and atrial fibrillation [26, 27]. Arterial stiffness is assessed using parameters such as augmentation index (AI) adjusted for a heart rate of 75 (AI-75) and pulse wave velocity (PWV).

**Fig. 6:**
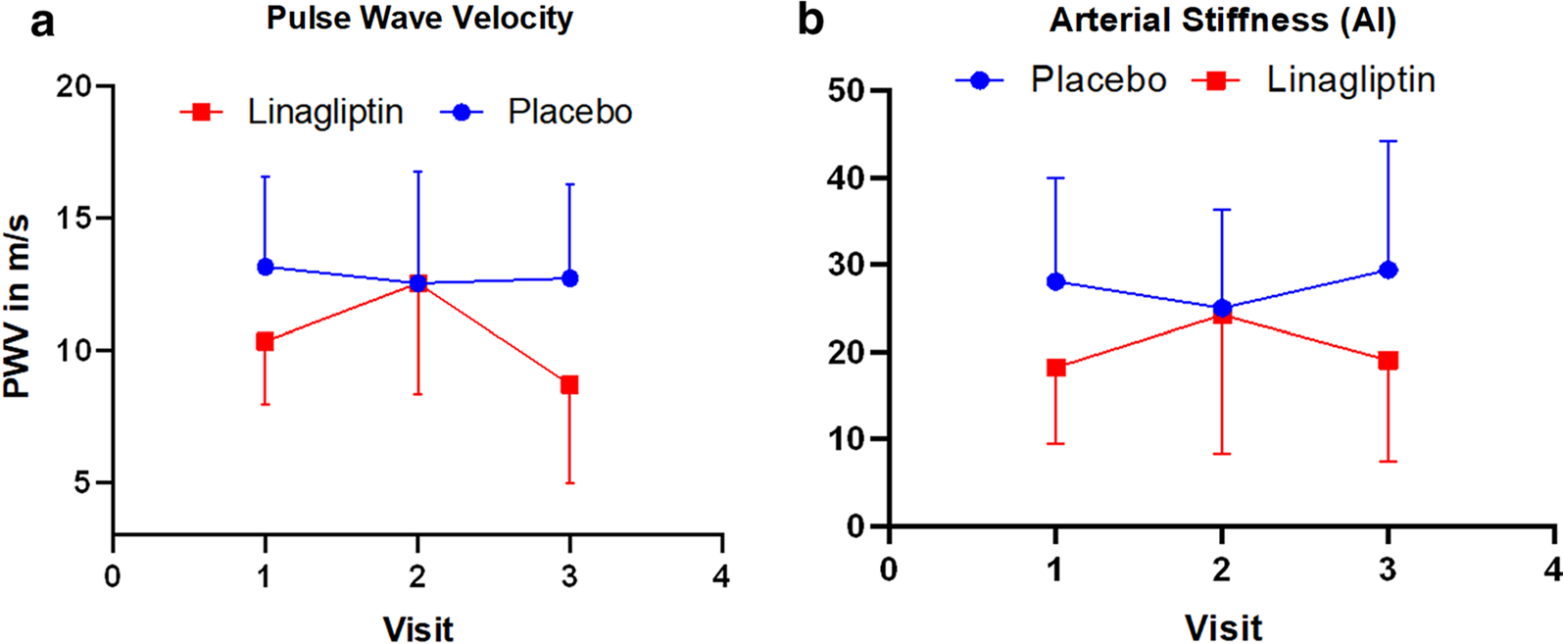
Arterial Stiffness Parameters. **a** Pulse wave velocity: at visit 1, the linagliptin group had lower PWV at a trend level of significance (p = 0.06). At visit 2, there was no difference (p = 0.91). At visit 3, the linagliptin group had significantly lower PWV (p = 0.03). The Linagliptin group increased PWV more from visit 1 to visit 2, and decreased more from visit 2 to 3, compared to the control group. **b** Pulse Wave Analysis: A similar pattern is seen here (comparing PWV with PWA). Error bars show the 95% confidence interval for the control group. There was not a significant effect of treatment group (p = 0.07), or of time (p = 0.98), but there was a significant group × time interaction (p = 0.02). The Linagliptin group increased more than placebo group from visit 1 to visit 2, but then rebounded back down more than the control group at visit 3

For PWV (Fig. 6a), At Visit 1, the linagliptin group demonstrated lower PWV at a trend level of significance (p = 0.06). At visit 2, surprisingly there was no difference (p = 0.91), however at visit 3, or at the end of the study, the linagliptin group had significantly lower PWV (p = 0.03), compared to placebo group. The linagliptin group showed significant decrease from visit 2 to 3, compared to the placebo group. No statistically significant changes in systolic blood pressure was noted between the groups.

AI-75 was found to be statistically significant along with augmentation pressure (AP) (Fig. 6b). There was no significant effect of treatment group (p = 0.07), or of time (p = 0.98), but there was a significant group × time interaction (p = 0.02). The linagliptin group increased more than placebo group from visit 1 to visit 2, but then reversed back down more than the control group at visit 3.

#### Adiposity

Body composition measurement showed no statistically significant change amongst the group throughout the visits. As expected, given short duration of the treatment the subjects were asked to maintain activity level as advised by American Diabetes Association (ADA) for healthy living. There was no statistically significant change in hip to waist ratio, body weight and body fat percent amongst the treatment and control group. As we know from prior study that physical activity, even for a short duration, can improve endothelial function and CD34+ circulating progenitor cells in patients with endothelial dysfunction [28].

#### Resting metabolic rate

Interestingly there was a large baseline difference in Basal Metabolic Rate amongst the treatment 1868 ± 293 and control group (1476 ± 448) but the change between the groups in this parameter was not clinically significant (p < 0.87).

## Discussion

### Primary outcome: cellular

In this study we investigated the effect of DPP4 inhibitor linagliptin in addition to metformin and/or Insulin on CD34+ EPCs and CD34+ CD184+ cells as a marker for vascular endothelial function. CD184 is the marker for SDF1a receptor. We also monitored gene expression of CD34 negative cells. The latter will be reflective of a population similar to mononuclear cells (MNC) and can give indication of general health of hematopoietic cells. The subjects recruited in this study had T2DM and have established CKD, similar to, established cardiovascular disease. As discussed in our previous study on saxagliptin, we used CD34+ cells as marker to identify EPCs [25]. Here we have shown that, treatment with linagliptin significantly increased (p = 0.02) the CD34+ EPC number as compared to placebo group. Similarly, CD34+ CD184+ve cells were increased significantly from visit one to visit two and then show a persistence in trend showing increase from visit 2 to 3. Taken together our observation of an increase in CD34+ EPCs and CD34+ CD184+ cells (Fig. 1) in linagliptin group are similar to the results from a recent study looking into the effects of linagliptin alone on EPCs in T2DM subjects [20]. It has been reported that CD34+ cells from patient with T2DM have impaired chemotaxis response to SDF1α resulting in reduced vasculogenic potential [24, 29]. In addition, the increase in CXCR4 (CD184+) expression is correlated with the increased migratory response to SDF1-α of CD34 + cells in linagliptin group (Fig. 1c).

### Gene expression

In order to understand the genotypic effect of linagliptin and metformin on gene expression we did qPCR analysis on CD34 negative cells. These cells are primarily hematopoietic cells. Here, we observed significant increase in antioxidants (SOD2, catalase and glutathione peroxidase 1 or GPX1) in linagliptin group as compared to placebo (Fig. 2b). In support of this result, we also found significant increase in the endothelial markers and functional genes (PECAM1, VEGF-A and vWF and NOS3) in linagliptin group as compared to placebo group (Fig. 2a). These observations are in agreement with our previous published results suggesting patho-physiological role of ROS activation and therapeutic reduction in CD34+ve cells in diabetes [25]. Regarding CD34+, individuals with longer duration of T2DM exhibited reduced frequencies of circulating proangiogenic high aldehyde dehydrogenase CD34+ progenitor cells with primitive (CD133) and migratory (CXCR4) phenotypes [30]. Hence increased antioxidants found in CD34+ cells may promote anti-inflammatory angiogenic vascular regeneration.

### Secondary outcomes: clinical

There are several studies that shows positive effect of incretins (Glucagon like peptide, GLP-1) and incretin receptor agonist (GLP1 receptor agonists) on cardiovascular risk factors in T2 DM [31–33], even in patients with chronic heart failure and left ventricular dysfunction who do not have diabetes [34, 35]. DPP-4 inhibitors may have cardio-protective effects of their own, as they increase bioavailability of endogenous GLP-1. They improve blood flow and Nitrous Oxide bio-availability in endothelium. These are unique properties not demonstrated by other oral diabetic medications [34, 35]. The mechanism underlying these hemodynamic changes may be mediated by increased nitric oxide bioavailability but is not completely known. However, these beneficial effects may appear to be independent of glycemic reduction. Linagliptin specifically has been shown to be protective for both macrovascular and microvascular complications of diabetes via improvement in tissue remodeling associated with accumulation of CD34+ cells [36].

All subjects were on a stable dose of metformin (1–2 grams/day) or insulin for at least 3 months or greater. Actigraph energy monitor data analysis showed there was no difference in the average intensity of daily activity between the treatment and placebo groups. This indicated that no changes in any outcome measures in the linagliptin group can be attributed to just exercise. There are also few studies that show the effect of metformin on endogenous GLP1 [37, 38]. These pose the possibility of a limit to the effect of DPP-4 inhibitors and Incretin analogues on patients with concurrent metformin. More studies are needed, possibly on patients without metformin to clarify these effects. These also might explain the modest effect we noticed in this study and our last study with Saxagliptin.

### Arterial stiffness parameters

Arterial stiffness is a measure of compliance and contractility of one’s arteries, and their ability to constrict and dilate in response to changes in blood pressure. It is measured non-invasively by assessing pulse wave velocity (PWV) and pulse wave analysis (PWA) and has been noted to increase naturally with age.

PWV is measured as a velocity in m/s. Higher values of augmentation pressure (AP), augmentation index (AI), AI-75, and PWV are correlated to higher levels of arterial stiffness. PWV, in addition to PWA measures such as blood pressure and AI, have been found to be a predictor of increased CVD risk in the general population, and especially in those at an increased risk, such as patients with T2DM. Arterial stiffness, being a direct measure of the radial, carotid and femoral arteries, would be expected to change with significant alterations to the endothelium [19, 39, 40].

There was statistically significant difference noted in Augmentation Index-75, a measure of arterial stiffness, between the treatment and placebo group. There was no significant effect of treatment group (p = 0.07), or of time (p = 0.98), but there was a significant group x time interaction (p = 0.02). The Linagliptin group showed improved parameters of arterial stiffness compared to the placebo group if all data from visit 1 to visit 2 and visit 3 are considered (Fig. 6b). Interesting pattern can also be appreciated in Fig. 6a, of Pulse Wave Velocity, another measure of arterial stiffness. At Visit 1, the linagliptin group had lower PWV at a trend level of significance (p = 0.06). At visit 2, there was no difference (p = 0.91). At visit 3, the linagliptin group had significantly lower PWV (p = 0.03). The Linagliptin group showed increased PWV from visit 1 to visit 2, however decreased significantly more from visit 2 to 3, compared to the control group. Our study shows a reduction in arterial stiffness in the Linagliptin group, as seen through a reduction in AI-75. This was also seen with other DPP-4 inhibitors, sitagliptin and vildagliptin, which resulted in a reduction in AI-75 [41, 42]. Arterial Stiffness, as measured via AI-75 is a strong predictor of CVD in Type 2 Diabetes [42–44]. The reduction in AI-75 may be attributed to a multi-platform effect. DPP-4 inhibitors cause an increase in systemic incretin levels, which can cause a relaxation of the arteries via nitric oxide (NO) [42]. This could be attributed to a reduction in arterial stiffness. Also, the higher percentage of CD34+ve CXCR4 receptor +ve cells that was reported in our cellular analysis may indicate that EPCs are having a regenerative effect on the subjects’ arteries across the 12-week time-period.

The Saxagliptin study we conducted did not have arterial stiffness improvement (particularly PWV) as robustly as Linagliptin, despite this cohort being sicker with both T2DM and CKD. This is corroborated by de Boer et al. which had similar result with 26 weeks of treatment with Linagliptin [43]. Therefore, Linagliptin appears to have clinically relevant and important arterial stiffness reduction capability even more so another similar compound (saxagliptin) within the same class of medications (DPP4 enzyme inhibitors). Another modality to measure arterial stiffness is Flow Mediated Dilatation (FMD). Another study argued, where modality was FMD, linagliptin treatment in subjects with CAD and early T2DM did not improve endothelial function or arginine bioavailability [45]

Finally, DPP-4 inhibitors help patients achieve a better level of glycemic control noted even a relatively short period of intervention of 12 weeks.

### Physical parameters

There also was no change in waist or hip circumference measurements, which is consistent with other studies involving linagliptin and saxagliptin, although these studies did not have concomitant metformin therapy [43]. These parameters usually takes longer to show a response within a short period of 12 week duration which maybe too short to demonstrate bio-physical changes. Previous studies have shown involving mice have shown that treatment with DPP-4 inhibitors in hyperglycemic obese mice resulted in reduction in adiposity, both in body fat percentage and abdominal fat mass [41]. This was attributed to an increase in energy expenditure, which was measured via monitoring metabolic rate and food intake. The difference in change in weight between the treatment and placebo group was not statistically significant, alongside Resting Energy Expenditure.

Study such as CARMELINA was done on a similar cohort [46], with T2DM and CKD, randomized to either 5 mg Linagliptin or placebo added to standard baseline treatment. That study was endpoint driven and Linagliptin was found to be noninferior to placebo plus standard treatment. Based on our results, the positive CVD outcome for CARMELINA, may be attributed to better chemotaxis of CD34+ve cells and improvement in arterial stiffness. A real-world database analysis demonstrates that DPP-4 inhibitor therapy did not increase the overall risk of MACE (major adverse cardiovascular outcome) and renal outcomes compared to sulfonylureas [47].

Previous studies investigating DPP-4 inhibitor therapy mainly sitagliptin and vildagliptin, in a type 2 diabetes population found that there was no significant reduction in HbA1c values with treatment [42]. An interesting pattern can be appreciated in Fig. 5 (HbA1c). The strongest effect of all the variable is the effect on time on HbA1c (p < 0.005), which means HbA1c went down substantially in both the treatment and control group. Even though the effect of treatment on this variable was not significant, after the effect of time was accounted for, interesting trend pattern can be appreciated when it’s graphed. The Hba1c is relatively stable in the placebo group but has a clear downward trend in the treatment group. We found statistically significant difference in the LDL over HDL ratio (p < 0.04). Lower the number, better the clinical outcome because higher ratio means a combination of increasing LDL and decreasing HDL, both of which correlates with adverse clinical outcome. A better profile of LDL:HDL ratio may corroborate with the ability of Linagliptin to improve cardiovascular risk profile.

As one of the main inclusion criteria of this study was subjects with CKD, all the data were stratified between CKD with normal GFR (Stage I) and CKD with reduced GFR (Stage II and below, or GFR < 60). There was no difference in effect of the treatment on the parameters that was studied. When Microalbumin over Creatine ratio was graphed (Fig. 4), it can be appreciated that proteinuria has remained steady in placebo group whilst in Linagliptin group there was a transient rise followed by a drop with higher slope or gradient, compared to the placebo group. This relationship was not statistically significant.

Furthermore, function of CD34+ cells in diabetic wound healing is of research interest. Animal studies showed nanofiber-expanded human CD34+ cells heal cutaneous wounds in streptozotocin-induced diabetic rodents [48]. Another study showed CD34 + cells alter molecular pathways associated with diabetic retinopathy pathogenesis and preserve retinal vasculature [49]. Hence, it is of clinical interest we find ways and methods to increase CD34+ve cells in diabetic patients.

### Urine exosomes

To discern the effect of linagliptin on kidneys and particularly podocyte health, we also looked at three urinary exosome proteins, such as podocalxyin, Wilm’s Tumor (WT) and nephrin. These levels were compared to CD-9 and Alix, two exosome markers. Though our results did not give a statistical difference it clearly showed a trend of improvement in the levels of the urinary exosomal proteins in this T2DM+ CKD population. We believe urinary exosomes could be an important clinical modality to discern podocyte health.

### Summary discussion

Overall, we believe that cellular parameter such CD34+ progenitor cell study along with clinically relevant parameters such as arterial stiffness helps to evaluate a diabetes medication quite thoroughly. Based on our studies using exercise physiology, saxagliptin and linagliptin as interventions we believe along with few other investigators [9] that circulating endothelial progenitor cells can help assess and possibly predict future risk of adverse cardiovascular morbidity and mortality. Our study indicates certain positive aspects of linagliptin, compared to saxagliptin, such as increased CD34/CD184 cell numbers, lesser PWV, improved HbA1C and positive antioxidant gene expressions in blood derived cells.

### Limitations of our study

Limitations of our study may include the relatively short duration of 12-week Linagliptin therapy, which may have been inadequate to see significant changes in certain clinical and cellular parameters. This may have been because of the small sample size, and due to the difficulty in obtaining all cellular outcome measures, in some patients, due to low total CD34+ cell numbers. Further studies with a larger population and longer duration may be helpful to further define the mechanisms behind our findings.

## Conclusion

It could be concluded that when Linagliptin when added to subjects with T2 DMand CKD, along with metformin and/or Insulin, demonstrates a functional improvement of CD34+ Endothelial Progenitor Cell migratory function through increased CD34/CXCR4 positivity. We have also observed concomitant improvement in Arterial stiffness parameters along with improvement in lipid profile and HbA1C. Our urine exosome studies indicate a possible improvement of podocyte health with a 12-week Linagliptin therapy.

We believe CD34+ cells can act as a valuable biomarker for assessment of endothelial function, in a setting of diabetes and similarly urine exosome analysis can help elucidate and predict renal function improvement. These two biomarkers can help provide valuable clinical information leading to appropriate therapeutic intervention choices in a face of ever increasing number of subjects with type 2 diabetes mellitus.

## Data Availability

All associated data will be available to the public, as requested. Demography of subjects including detailed description of baseline characteristics and parameters have been included in Table 1.

## Appendix

### Inclusion criteria

1. Adults aged 30-70 years.
2. Diagnosis of type 2 diabetes within the previous 15 years using criteria of the American Diabetes Association.
3. Currently treated with a stable dose of Insulin, Metformin (1–2 g/day), or a stable combination of the two as therapy.
4. HbA1C between 6.5 and 10% (both inclusive).
5. BMI 25 to 39.9 kg/m^2^ (both inclusive).
6. CKD stages 1–3.

### Exclusion criteria

1. Implanted devices (e.g., pacemakers) that may interact with Tanita scale.
2. Previous coronary or cerebrovascular event within 6 months of screening or active or clinically significant coronary and/or peripheral vascular disease.
3. Low hematocrit (< 28 UNITS).
4. Pre-existing liver disease and/or ALT and AST > 2.5X’s UNL.
5. CKD stage 4 and up.
6. History of pancreatitis, or cancer (except basal cell carcinoma and cancer that is cured or not active or being treated in the past 5 years).
7. Statin use started or dose change in the last 3 months.
8. Use of anti-diabetic medication other than Metformin, or Insulin.
9. Use of consistent long-term steroid medication in the last 3 months (oral, inhaled, injected).
10. Systolic BP > 140 mmHg and Diastolic BP > 90 mmHg.
11. Active wounds or recent surgery within 3 months.
12. Inflammatory disease, or chronic current use of anti-inflammatory drugs within the last 3 months.
13. triglycerides > 450 mg/dL.
14. untreated hyper/hypothyroidism.
15. Auto antibody confirmed type 1 diabetes.

Additionally, patients who are active smokers, patients who are pregnant, nursing women, and post-menopausal women who are on hormone replacement therapy will be excluded.

Patients on low dose oral contraceptives will be allowed to participate as these formulations contain lesser amount of estrogens.

## Abbreviations

AI: Augmentation Index
AI-75: Augmentation index adjusted for a heart rate of 75
AP: Augmentation pressure
AS: Arterial stiffness
BCl-2: B-cell lymphoma 2
BMI: Body mass index
BUN: Blood urea nitrogen
CASP-3: Caspase 3
CAT: Catalase
CDKN1A: Cyclin-dependent kinase inhibitor 1A
CD34: Progenitor marker
CFU: Colony forming units
CVD: Cardiovascular disease
CXCR4: C-X-C Motif chemokine receptor 4
DBP: Diastolic blood pressure
DPP-4 Inhibitors: Dipeptidyl peptidase-4 inhibitors
EDN-1: Endothelin 1
eGFR: Estimated glomerular filtration rate
ELISA: Enzyme-Linked Immunosorbent Assay
eNOS: Nitric oxide synthase 3 (endothelial cell)
EPCs: Endothelial progenitor cells
FFM: Fat free mass
GAPDH: Glyceraldehyde-3-phosphate dehydrogenase
GLP1: Glucagon like peptide 1
GPX1: Glutathione peroxidase 1
HbA1C: Hemoglobin A1C, glycated hemoglobin test
IGF1: Insulin-like growth factor 1
IL-6: Interleukin 6
kg: Kilograms (weight)
lbs: Pounds (weight)
MNCs: Mononuclear cells
PECAM1: Platelet and endothelial cell adhesion molecule 1
PWA: Pulse wave analysis
PWV: Pulse wave velocity
qRT-PCR: Quantitative reverse transcriptase polymerase chain reaction
REE: Resting energy expenditure
SBP: Systolic blood pressure
SDF1α: Stromal cell-derived factor-1α
SOD1, SOD2: Super oxide dismutase 1 & 2
TBW: Total body water
TNFα: Tumor necrosis factor α
TP53: Tumor protein p53
VEGF: Vascular endothelial growth factor
VEGFA: Vascular endothelial growth factor A
VEGFR2: Vascular endothelial growth factor receptor 2
VO2: Maximal oxygen consumption
18S: 18S ribosomal RNA
